# Nutritional influences on enzyme activities in saliva of Asian and African elephants

**DOI:** 10.1007/s00360-021-01378-6

**Published:** 2021-07-07

**Authors:** Carolin Boehlke, Sabrina Schuster, Lucas Kauthe, Oliver Zierau, Christian Hannig

**Affiliations:** 1 Institute of Zoology, Molecular Cell Physiology and Endocrinology, TU Dresden, Zellescher Weg 20b, 01062 Dresden, Germany; 2 Policlinic of Operative and Pediatric Dentistry, Faculty of Medicine ‘Carl Gustav Carus’, TU Dresden, Fetscherstraße 74, 01307 Dresden, Germany

**Keywords:** Elephant, Salivary enzymes, Amylase, Lysozyme, Peroxidase, Nutrition

## Abstract

Asian and African elephants show morphological adaptations to their ecological niche including the oral cavity. Variety and preferences of forage plants differ between both herbivorous elephant species. Diet can affect salivary enzymes. Asian elephants were shown to have a higher salivary amylase activity than African elephants. Species-specific differences were presumed to be influenced by feeding during collection procedure. This study aimed to determine the influence of feeding on enzyme activities in saliva of both elephant species to differentiate from species-specific effects. Additionally, season and housing conditions on salivary enzyme activities in non-fed elephants of both species were investigated. Salivary amylase (sAA), lysozyme (sLYS) and peroxidase (sPOD) activity were measured photometrically or fluorometrically. Results of this study reinforce previous observations of higher basic sAA activity in Asian elephants compared to African elephants. Salivary LYS and sPOD activity showed neither species-specific nor housing-specific differences. Independent from season, most elephants of both species revealed a lack of or low sPOD activity. Feeding caused a temporary decrease of sAA, sLYS and sPOD activity in both elephant species kept in four of eight tested zoos. Furthermore, sAA activity in Asian elephants was higher and sLYS activity lower in Spring than in Autumn. This study summarizes that sAA and sLYS are components of Asian and African elephant saliva in an active conformation in contrast to sPOD. Diet varying between season and zoos might influence sAA and sLYS activities primarily in Asian elephants but temporary low effects suggest sufficient buffer capacity of elephant saliva of both species.

**Supplementary Information:**

The online version contains supplementary material available at 10.1007/s00360-021-01378-6.

## Introduction

Nowadays, the order Proboscidea is only represented by three still living species of elephants, i.e. the African bush elephant (*Loxodonta africana, AF*), the African forest elephant (*Loxodonta cyclotis*), and the Asian elephant (*Elephas maximus, AS*). The two elephant genera descended from the 55-mya-old *Phosphatherium*, whereas *Eritherium azzouzorum* is considered the oldest and most primitive elephant relative (Gheerbrant 2009). Approximately 7.6 million years ago, the lineage of ancestors of African elephants diverged from the Elephantidae lineage leading to mammoths and Asian elephants (Rohland et al. 2007). However, mammoths and Asian elephants diverged approximately 6.7 million years ago (Rohland et al. 2007). The genome of both elephant species is similar, i.e. their sequences align to approximately 94% (Dastjerdi et al. 2014). All recent elephant species, unlike some of their extinct relatives, occur in the tropics or adjacent areas. At present, the natural habit of the African bush elephant is sub-Saharan Africa (Blanc et al. 2007), whereas the Asian elephant lives in South and Southeast Asia (India, Bangladesh, Thailand, Myanmar, Cambodia) (IUCN 2008).

The elephants species are herbivorous generalists but less selective than smaller herbivores (Codron et al. 2012; Owen-Smith and Chafota 2012; English et al. 2014). They consume a wide variety of plants depending on seasonal and regional abundance and become unselective in dry season when food availability is sparse (Owen-Smith and Chafota 2012; Shrader et al. 2012). African elephants were shown to consume higher percentages of leaves in wet season in contrast to the dry season when almost only stem, bark, and root tissues are fed (Owen-Smith and Chafota 2012). Grazing and browsing varies depending on the habitat and the season (Guy 1976; Koch et al. 1995; Ullrey et al. 1997; Codron et al. 2006). In wet season, African elephants as well as Asian elephants tend to graze and they spend more time on feeding in contrast to the dry months when feeding less long characterizing them rather as browser (Wing and Buss 1970; De Boer et al. 2000; Mohapatra et al. 2013; Greene et al. 2019). Studies summarized that seasonal deteriorations in grass quality might lead to browsing by the Asian elephant (English et al. 2014) and African elephants (Owen-Smith and Chafota 2012).

Both elephant species are able to exploit a wide range of plant parts due to their large size coupled with hindgut digestion (Owen-Smith and Chafota 2012). Diet of both elephant species ranges from large trees to small herbs depending on the season (Guy 1976; Kabigumila 1993), i.e. it comprises fruits, bulbs, and roots in addition to grasses, forbs, shrubs, and trees (Ullrey et al. 1997). Preferences of these dietary components vary between elephant species. Several authors summarized that diet of some forest elephant species is predominated by browse when being present in large quantities (English et al. 2014). However, grasses account for the largest share of the diet of forest elephants in Cameroon independent from season (Tchamba and Seme 1993; English et al. 2014). English et al., (2014) also described that larger herbivores like elephants may respond to plant size rather than responding only to chemical or structural composition of food plants during food selection (Vivås et al. 1991; Wilson and Kerley 2003).

African elephants consume a diverse number of plant species (36–133) (Guy 1976; Kabigumila 1993) and prefer trees with higher protein and lower fibre concentrations irrespective of season (Ward et al. 2017). A study on African elephants showed no relationship between the vigor of plant species, i.e. growth rate and nutritional composition, and its consumption preferences (Makhabu et al. 2006; English et al. 2014). Furthermore, it was shown that soil quality and associated leaf quality of plants might affect foraging decisions of African elephants in the dry season, but not in the wet season (Ward et al. 2017). However, a study showed that diet selection of African elephants is less driven by content of crude protein, in-vitro digestibility, and total polyphenol concentration (Schmitt et al. 2020b). Odor-directed foraging preferences help African elephants, to recognize e.g. specifically emitted potentially toxic volatile secondary plant metabolites that serve as antifeedants or digestion inhibitors prior to ingestion of food items, and therefore appear to be a better indicator for avoidance of food items (Bryant et al. 1991; Owen-Smith and Chafota 2012; Schmitt et al. 2020b). Certain woody species, e.g. species of Combretaceae are known to contain high contents of total polyphenols; these were partly or entirely rejected by African elephants irrespective of season, although they are fed by other herbivores, e.g. giraffe, kudu, and impala (Makhabu et al. 2006; Owen-Smith and Chafota 2012).

Asian elephants feed on 95–112 different plant species, but about 85% of the whole food intake accounts for only 25 of these species (Sukumar 1989; De Boer et al. 2000). Furthermore, Asian elephants are known to select food highly seasonal and relating to their protein content (Guy 1976; Joshi and Singh 2008; Mohapatra et al. 2013) and might ingest a higher proportion of grasses than African elephants (Cerling et al. 1999; Clauss et al. 2007) but varies seasonal (Sukumar 1989). They also achieve higher digestion coefficients for dry matter, hemicellulose, and cellulose than African elephants when fed comparable diets (reviewed in Greene et al. 2019). Investigations on Bornean elephants (*Elephas maximus borneensis*) showed a consumed plant range of 182 plant species without preferences for species with larger or smaller growth forms (English et al. 2014). Unlike other forest elephants, the Bornean elephant prefers species from the Poaceae rather than other plant types including gingers, palms, lianas, and woody trees (English et al. 2014). These differences between both elephant species might reflect their adaptability to different ecological niches, e.g. within the oral cavity.

Salivary enzymes are, as expected, intensively studied in humans. Most relevant salivary enzymes for protective and digestive functions are amylase (sAA), lysozyme (sLYS), and peroxidase (sPOD) (Kaufman and Lamster 2000; Humphrey and Williamson 2001; Nater and Rohleder 2009). The most abundant protein in human parotid saliva is the salivary α-amylase which is also known as one of the key digestive enzymes in saliva of many other mammals (Noble 2000; Nater and Rohleder 2009; Carpenter 2013; Boehlke et al. 2015). The enzyme catalyzes polysaccharide digestion by cleaving their α-(1,4)-glycosidic bonds. Salivary lysozyme cleaves peptidoglycans of bacterial cell walls whereby it has an antibacterial function based on its muramidase activity (Laible and Germaine 1985; Wang and Germaine 1993). Salivary peroxidase serves as an important defense system of the oral cavity against bacteria. Due to its antioxidative and antibacterial function (Battino et al. 2002; Ihalin et al. 2006) bacterial colonization in the oral cavity can be prevented (Steele and Morrison 1969; Björck et al. 1975; Pruitt and Adamson 1977).

Salivary enzymes can be affected by diet. Polyphenols such as tannins might inhibit sAA due to their binding affinity shown in humans (Butler 1989; Kandra et al. 2004; Serrano et al. 2009). As mentioned before, these secondary plant metabolites serve as natural antifeedant. When tannin rich food items are fed, a secretion of proline-rich proteins in the oral fluids up to 45% is induced (Butler 1989; Mau et al. 2009), which are able to bind tannins and other polyphenols (de Freitas and Mateus 2001). Furthermore, other proteins with a high tannin-binding affinity have been found in saliva of browsing megaherbivores (Schmitt et al. 2020a). This physiological mechanism was shown to help many browsers and intermediate feeders, including elephants, to tolerate a portion of tannins (Schmitt et al. 2016, 2020a). A major source of tannins in dietary components are brans of grains, legumes, nuts, and fruits, especially their peel and the unripe fruit flesh (Wrangham and Waterman 1983) in contrast to low amounts in vegetables, corn, rice and wheat (Deshpande et al. 1986; Serrano et al. 2009). It was shown that condensed tannins are more abundant in food than hydrolysable tannins and fed plants contain no more than approximately one to two percent condensed tannins (Butler 1989). In elephants´ natural habitat, particularly foliage and leaves of different plant species contain polyphenols (Cooper and Owen-Smith 1985; Robbins et al. 1987; Owen-Smith and Chafota 2012; Shrader et al. 2012). As mentioned before, African elephant in northern Botswana tend to avoid certain legumes and woody species that contain polyphenols especially in leaves rather than in roots (Owen-Smith and Chafota 2012). Tannin-rich dietary components are partially also fed to elephants kept in zoos, e. g. oak and poplar branches, legumes and unripe fruits. When less food is available, some herbivores show possible adaptions and even preferences for tannin-rich food items (reviewed in Butler 1989). It was shown that elephant saliva might have evolved an incorporation of bypassing negative effects and partial neutralization of plant secondary metabolites by the tannin-binding affinity (Schmitt et al. 2016).

Certain other fruits and vegetables fed to elephants in zoos might contain further inhibitory ingredients for sAA activity, as it has been shown for ascorbic acid (Purr 1934; Abell et al. 1998), fisetin (Sales et al. 2012) to be found in apples, grapes, and kiwis (Adhami et al. 2012), in addition to luteolin which can be found in carrots (Lopez-Lazaro 2009). Partly eaten starch-rich food by elephants in zoos and their natural habitat can also influence sAA activity as it was shown in humans. Starch-rich diet might affect amylase gene (*AMY1*) copy number, which can lead to higher sAA activity (Perry et al. 2007; Mandel et al. 2010). sLYS activity can be inhibited by imidazole and indole derivatives, which can be found in wheat and corn (Shinitzky et al. 1966) as well as by sorbitol from, e.g. pome fruit varieties (Mäkinen and Söderllng 1980; Kim et al. 2015) and tannins (Green 1995). Flavonoids were shown to increase sPOD activity (Gau et al. 2018), whereas xylitol, sorbitol and cyanides occurring in apples can inhibit the enzyme (Kim et al. 2015) as well as polyphenols (Hannig et al. 2008).

Until now, composition of Asian and African elephant saliva has been rarely studied (Raubenheimer et al. 1988; Menargues et al. 2012; Marcilla et al. 2012; Illera et al. 2014; Edwards et al. 2019; Plangsangmas et al. 2020; Hambrecht et al. 2020). This study is based on a previous comparative investigation of salivary enzyme activities in Asian and African elephants, which collected saliva only once a day (Boehlke et al. 2016). Because of the one-time collection of saliva, no conclusions could be drawn concerning to diurnal rhythm of enzymes in elephant saliva of both species based on this previous study. Furthermore, it was unknown whether observed variations in sAA activity between both elephant species were species-specific or based on other effects, e.g. feeding. Furthermore, all elephants independent from species revealed an unexpectedly low sPOD activity, which possibly was caused by inconsistency during and/or feeding immediately before sample acquisition (Boehlke et al. 2016).

Therefore, this study attached importance to a standardized saliva collection procedure for both elephant species, which were kept in seven German zoos. The aim was to determine influence of feeding condition in both elephant species on salivary enzyme activity (I), i.e. sobriety and feeding to differentiate from species-specific effects. Intake of food might have an immediate effect on enzyme activities (1). Furthermore, effect of housing condition (II) and season (III) on salivary enzyme activities were investigated in non-fed Asian and African elephants. Zoo-specific (2) and seasonal (3) variety of diet might cause different enzyme activities in both elephant species kept in different zoos. This study aims to contribute insights into species-specific enzyme activity profiles of herbivores combined with investigations of nutrition as one influencing factor on salivary enzyme activities.

## Material and methods

### Specimens

Saliva was collected as described below from nine male and 18 female Asian elephants as well as from three male and 13 female African elephants (total *n* = 43) kept in different zoos in Germany (Table 1).

**Table 1:** Summary of information of tested elephants from different German zoos

	Zoological institution	Collection date		Sex	Year of birth	ID	Condition at collection time
Asian Elephants (*Elephas maximus*)	Zoo Münster	13.03.18	–	♀	1992	asM1	Non-fed: 08:30 Fed: 10:30 3 h > fed: 14:00
			27.2.19	♀	1967	asM2	
				♀	1996	asM3	
				♀	1966	asM4	
				♀	1966	asM5	
	Kölner Zoo	14.03.18	28.11.18	♀	1984	asK1	Non-fed: 08:30 Fed: 9:30 3 h > fed: 15:30
				♀	1980	asK2	
				♀	2007	asK3	
				♀	1989	asK4	
				♀	1988	asK5	
		–		♂	1999	asK6	
		–		♂	2011	asK7	
		–		♀	1994	asK8	
		–		♀	1990	asK9	
		–		♂	2016	asK10	
		–		♂	1969	asK11	
		–		♀	2006	asK12	
		–		♀	2012	asK13	
	Zoo Heidelberg	06.03.18	17.8.18	♂	2005	asHD1	Non-fed: 08:00 Fed: 10:00 3 h > fed: 14:30
				♂	2011	asHD2	
		–		♂	2008	asHD3	
		–					
				♂	2005	asHD4	
	Tierpark Berlin	27.03.18		♂	2016	asTB1	Non-fed: 10:30 Fed: 11:45 3 h > fed: 15:25
				♀	2008	asTB2	
				♀	1980	asTB3	
				♀	1980	asTB4	
				♀	1973	asTB5	
African elephants *(Loxadonta africana)*	Zoopark Erfurt	22.03.18	28.8.18	♂	2005	afE1	Non-fed: 07:25 Fed: 11:25 3 h > fed: 13:30
				♀	1971	afE2	
				♀	1995	afE3	
				♀	2003	afE4	
	Tierpark Berlin	27.03.18		♂	2007	afTB1	Non-fed: 10:30 Fed: 11:45 3 h > fed: 15:25
				♀	1971	afTB2	
				♀	1981	afTB3	
				♂	1985	afTB4	
	Zoo Dresden	18.04.18	06.11.18	♀	1995	afDD1	Non-fed: 07:20 Fed: 11:00 3 h > fed: 14:40
				♀	1996	afDD2	
				♀	1990	afDD3	
	Elefantenhof Platschow	04.05.18		♀	1984	afEP1	Non-fed: 09:45 Fed: 10:10 3 h > fed: 18:00
				♀	1988	afEP2	
				♀	1985	afEP3	
				♀	1984	afEP4	
				♀	1980	afEP5	

“Non-fed” indicates saliva samples from non-fed elephants, “fed” shows that elephants were fed immediately before saliva collection. Third saliva sample was collected 3 h after elephants were fed “3 h > fed”

### Saliva collection

Three to five saliva samples were collected from each Asian and African elephant at each sampling time, which were pooled to achieve a sufficient saliva volume using Salivette® (Sarstedt, Nümbrecht, Germany), respectively. The absorbing material was a synthetic swab. All Asian and African elephants were trained in their daily routine to respond to different commands, including the command to open their mouth, which allowed relatively easy excess to saliva. Saliva sampling was performed by the responsible animal caretaker of each zoo. Saliva was collected by wiping the absorbent material under the tongue as well as left and right to the tongue to ensure mixed saliva of different salivary glands for at least 30 s. The elephants of both species were in no way forced to participate in saliva collection, but participated voluntarily. Accordingly, the animals did not suffer or were stressed by the collection procedure. Due to this fact, the saliva collection procedure was not evaluated as an animal experiment in accordance with the institutional Animal Care and Use Committee nor by the German law.

#### Seasonal influence

The whole sampling procedure was performed twice a year, i.e. in Spring and Autumn. Saliva in Spring was collected in zoos between February and May, and saliva in Autumn was sampled in August and November. Asian elephants kept in Zoo Münster were sampled twice in Spring, i.e. in consecutive years. Seasonal influences on enzyme activities were tested on saliva samples of Asian elephants from Zoo Heidelberg and Kölner Zoo and on saliva of African elephants from Zoo Dresden and Zoopark Erfurt (Table 1).

#### Impact of feeding condition

In both seasons, saliva of elephants of both species was collected three times a day in each zoo, respectively. The first sample was taken in the morning from 7am to 10am, before Asian and African elephants were fed. In almost all zoos both species of elephants were not fed (non-fed), except for Asian and African elephants from Tierpark Berlin and African elephants from Elefantenhof Platschow, as they were fed at 7 am. Therefore, first saliva in both zoos was collected at least 3 hours later without food availability for both elephant species during this time. After saliva collection, Asian and African elephants were rewarded by dried wheat rolls, fruits and vegetables zoo specifically. Hay and branches of different plant species, which are described below (Table 2), were also available until the second saliva collection. The second saliva sample was collected between 10 and 12 am, 15 min after both elephant species were fed by a mixture of concentrated feed, i.e. pellets and oat or wheat bran (fed, Tables 1, 2). The third saliva collection was performed in the afternoon between 1:30 pm and 3:30 pm (3 h > fed), except for Elefantenhof Platschow, where saliva was collected at 6 pm. Between the second and the third saliva collection, hay and branches ad libitum were offered to both elephant species. During saliva sampling Asian and African elephants were not rewarded except during the third saliva collection with dried wheat rolls [all except Elefantenhof Platschow and Kölner Zoo (Spring)], by apples (Elefantenhof Platschow, Tierpark Berlin) and bananas (Elefantenhof Platschow, Zoo Dresden). Different time periods of collecting saliva resulted because of management reasons and daily routine of animal caretaker of each zoo. Therefore, in Tierpark Berlin, no third saliva collection in the afternoon of African elephants was possible.

**Table 2:**
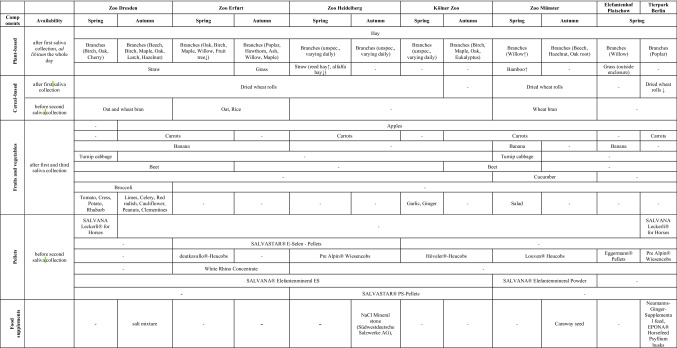
Dietary components of elephants from different zoos in Spring and Autumn

Upward arrows (*↑*) indicate high amount of food component, in contrast to downward arrows (↓), which show low amount of dietary component

### Nutrition

Nutrition of Asian and African elephants was zoo specific. In almost all zoos, carrots, apples and dried wheat rolls were offered at least once a day to elephants (Table 2). Availability of other fruits and vegetables that were fed to Asian and African elephants differed depending on the zoos (Table 2). Hay and branches were available nearly the whole day in all zoos. Offered plant species differed zoo specifically, but common fed genera were oak (*Quercus* spec.), birch (*Betula* spec.), maple (*Acer* spec.), poplar (*Populus* spec.) and willow (*Salix* spec.) (Table 2). SALVANA® Elefantenmineral ES were fed to both species of elephants in almost all zoos, except Tierpark Berlin and Elefantenhof Platschow. SALVASTAR® PS-Pellets were only available for Asian elephants kept in Kölner Zoo and Zoo Heidelberg. Hay pellets were fed in all zoos, but ingredients differed depending on manufactured company. White Rhino Concentrate pellets were only fed to African elephants in Zoopark Erfurt. SALVASTAR® E-Selen-Pellets were only available for African elephants kept in Zoopark Erfurt and Asian elephants in Zoo Heidelberg.

Nutrition varied between Spring and Autumn. Hay differed in composition of dried plants (not specifiable) as well as species of branches (Table 2). In Autumn, fresh grass was offered to African elephants only in Zoopark Erfurt and eucalyptus in Kölner Zoo, whereas bamboo was only available in Spring for Asian elephants in Zoo Münster. However, variety of fruits and vegetables slightly differed seasonal as well as food supplements fed to both elephant species. Offered pellets almost not differed between Spring and Autumn (Table 2).

### Determination of enzyme activity

After collection, all samples were stored on ice until centrifugation (~ 4 °C, 20 min, and 4.000 rpm). After centrifugation, saliva which was collected at the same time was pooled and aliquoted for each elephant and stored at − 80 °C at the Institute of Zoology, Dresden until analysis. The analysis of sAA, sLYS and sPOD was performed in triplicate for each saliva sample.

For the measurement of sAA activity, the low-molecular-weight substrate 2-chloro-4-nitrophenyl-4-*O*-β-*d*-galactopyranosylmaltotrioside (GalG_2_CNP) was used as described previously (Hannig et al. 2004). Briefly, this trisaccharide is linked via an α-glucosidic bond to the chromophore 2-chloro-4-nitrophenol. Hydrolyzation of GalG_2_CNP by sAA yields in 2-chloro-4-nitrophenol (CNP) (Morishita et al. 2000) at a constant rate without a lag phase, which can be determined photometrically at 405 nm.

The investigation of sLYS activity was performed fluorometrically using the hydrolysis of fluorescein-labelled *Micrococcus lysodeicticus* (EnzCheck Lysozyme assay kit; E-22013, Molecular Probes, Leiden, The Netherlands) as described previously (e.g. Vray et al. 1980).

For the investigation of sPOD activity, the fluorogenic 2′,7′-diacetlchlorofluorescin (LDCF) was used. In the presence of peroxidase and hydrogen peroxide, the substrate is converted to the fluorescing dichlorofluorescin (DCF) as described previously (e.g. Black and Brandt 1974). The sensitivity of the assay was enhanced by thiocyanate (Proctor and Chan 1994).

### Statistical analyses

Analyses were run in SPSS 25.0 (IBM Corporation, Chicago, Illinois, USA). Normal distribution of sAMY, sLYS and sPOD activities was tested via Kolmogorow–Smirnow test. To investigate age-related effects on different salivary enzymes, we ran Spearman (SP) and Pearson (P) correlations. Differences of enzyme activities between elephants of each species which are kept in different zoos were evaluated via one-way ANOVA and GT2-Hochberg post hoc test, respectively. For assessment of influence of elephant’s food intake on enzymes activities, Wilcoxon signed ranks test (data not normally distributed) or paired sampled *T* test (data normally distributed) was used. Differences in salivary enzyme activity between Asian and African elephant from Tierpark Berlin were investigated via not paired sample *T* test. Salivary enzymes activities of saliva samples of Asian and African elephants collected in Spring and Autumn were evaluated via one-way ANOVA and GT2-Hochberg post hoc test. The significance level was defined as *p* < 0.05 for all used tests. Zero values for enzyme activity (0 U/ml) were considered in the same way as other values during statistical analyses, indicating a very low or no detectable enzyme activity.

## Results

Salivary amylase and lysozyme were measurable in all saliva samples of both elephant species. In contrast, independent from season most elephants of both species showed no or low salivary peroxidase activity (see supplement Table 8). Higher sPOD activity was only observed in saliva, which was collected from three Asian elephants from Kölner Zoo in Spring (Table 3, see supplement Table 7).

**Table 3:** Summary of results for salivary amylase (sAA), lysozyme (sLYS) and peroxidase (sPOD) activities of Asian and African elephants

Species	Zoo	Condition	Enzyme activities								
			sAA			sLYS			sPOD		
African elephant (*Loxodonta africana*)	Elefantenhof Platschow	non-fed_fed	↓			↓			–		
		fed_3h > fed	↑			–			–		
	Zoopark Erfurt	non-fed_fed	–			(↓)			–		
	Tierpark Berlin	non-fed_fed	–	non-fed_AS > non-fed_AF		(↓)	non-fed_AS = non-fed_AF		–	non-fed_AS = non-fed_AF	non-fed_AS = non-fed_AF
	All	Seasonal difference	Spring = Autumn =			Spring = Autumn =			Spring = Autumn =		
	Age		Old = young =			old = young =			old = young =		
Asian elephant (*Elephas maximus*)			Old↑ young↓			old = young =			old = young =		
	All	Seasonal difference	Spring ↑ Autumn ↓			Spring = Autumn =			Spring = Autumn =		
	Tierpark Berlin (TP)	non-fed_fed	–	non-fed_AS > non-fed_AF	non-fed_TP > non-fed_K	(↓)	non-fed_AS = non-fed_AF	non-fed_TP = non-fed_K	-		non-fed_TP = non-fed_K
	Kölner Zoo (K)	–	–			–			–		
	Zoo Heidelberg	fed_3h > fed	↑			–			–		
	Zoo Münster	fed_3h > fed	–			–			**↑**		

The feeding condition “non-fed” indicates non-fed elephants, “fed” shows that elephants were fed immediately before saliva collection and “3 h > fed” indicates that saliva sample was collected 3 hours after elephants were fed. An underscore “_” between feeding conditions displays that measured enzyme activities were compared. Upward “↑” and downward “↓” arrows indicate an increase or higher as well as a decrease or lower enzyme activity. Arrows in brackets “(↑↓)” show a changing trend of enzyme activity. An equal sign “ = ” indicates an equal and “ > ” a higher enzyme activity. A dash “– “ displays no change or effect on enzyme activity

Influence of sex on sAA, sLYS and sPOD activities was not evaluated due to unbalanced number of tested male and female elephants of both species (Table 1). First, the effect of age on the salivary enzymes was analyzed. Age was positively correlated with sAA activity in non-fed Asian elephants in Spring and in Autumn in contrast to sLYS and sPOD activity (Table 3, see supplement Table 8). Older Asian elephants showed higher sAA activity independent from season (Table 3, see supplement Table 8). In non-fed African elephants, age was not correlated with any of the enzyme activities neither in Spring nor in Autumn (Table 3, see supplement Table 8).

**Fig. 1:**
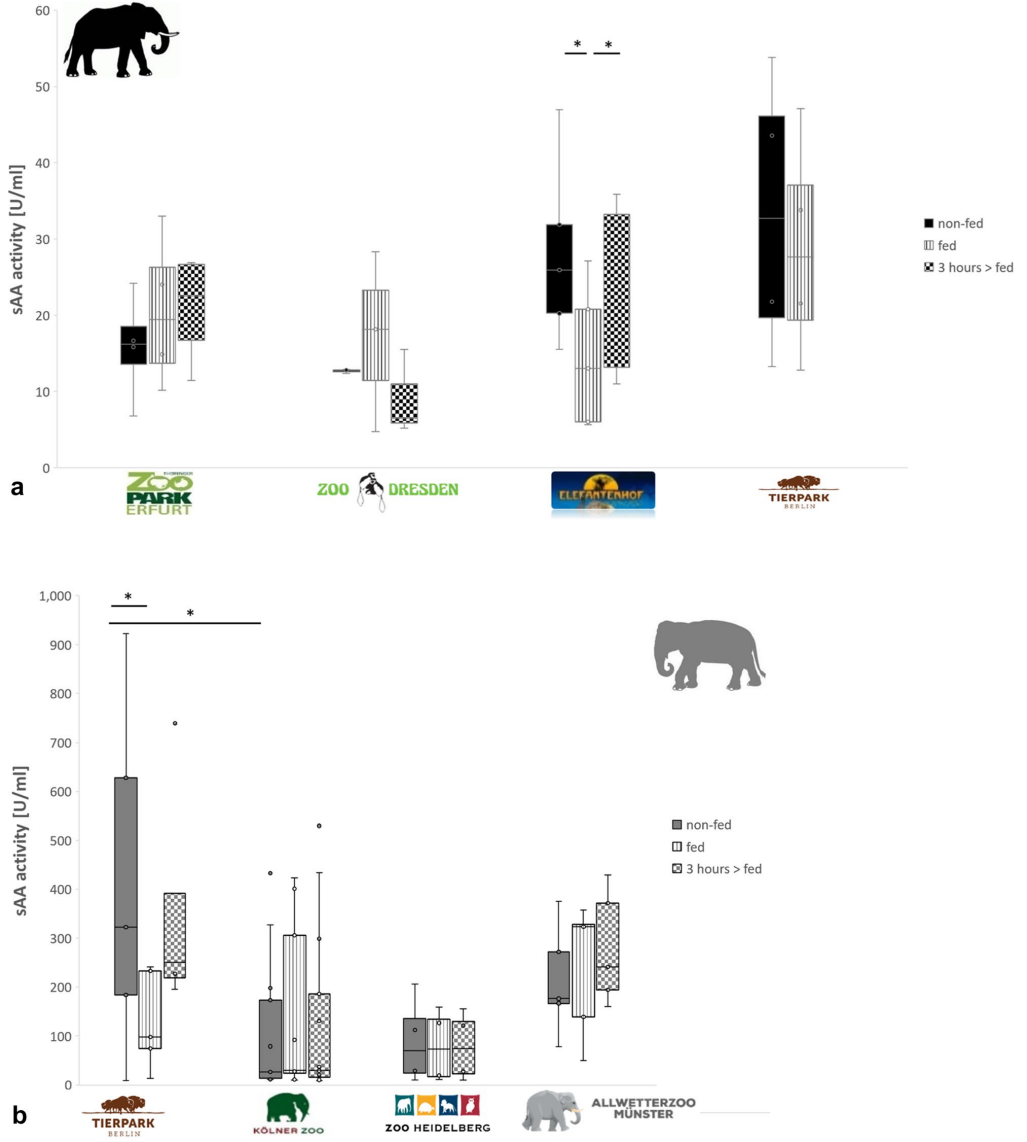
Salivary amylase activity of elephants at different feeding conditions from different zoos. **a** sAA activity of non-fed and fed African elephants as well as three hours after they were fed is shown in black boxes. **b** sAA activity of non-fed and fed Asian elephants as well as three hours after they were fed is shown in grey boxes. The boxes illustrate the 25th and 75th percentiles, bars show medians. Black or grey filled boxes indicate saliva samples from non-fed elephants. Boxes with vertical stripes indicate saliva samples of fed elephants. Boxes with dots indicate saliva samples of elephants 3 hours after they were fed. Separated dots show outliers. The significance is displayed by *(*p* < 0.05), which illustrates a significant difference

Analyses of enzyme activity of both elephant species kept in the same zoo (Fig. 1 a, b) showed higher sAA activity in Asian than in African elephants from Tierpark Berlin (Table 3, *T* = − 3.030; *df* = 5; *p* = 0.029, mean difference = 545.30 U/ml). In addition, no differences in sLYS and sPOD activity in Asian and African elephants from Tierpark Berlin were observed (sLYS: *T* = 1.827; *df* = 5; *p* = 0.127, mean difference = 66.84 U/ml; sPOD: *T* = − 2.076; *df* = 5; *p* = 0.093, mean difference = -4.971 U/ml).

### (I) Influence of feeding condition on salivary enzyme activity

Saliva of both elephant species, non-fed, was collected three times a day after elephants were fed and circa three hours after feeding in the afternoon. In contrast to other African elephants, averaged sAA activity of African elephants kept in Elefantenhof Platschow decreased significantly after they were fed (non-fed: 28.11 U/ml, fed: 14.52 U/ml; Table 3, see supplement Table 6, Fig. 1a). Within three hours after being fed, averaged sAA activity increased circa at the same level when they were not fed (3 h > fed: 25.16 U/ml, Table 3, see supplement Table 6). Variations of sAA activity of African elephants from Zoo Dresden were lower when they were not fed in contrast to that after feeding. Feeding Asian elephants showed a decrease in averaged sAA activity in Tierpark Berlin (non-fed_413.18 U/ml, fed_132.39 U/ml, Table 3, see supplement Table 6), but there were no differences found in averaged sAA activity between non-fed condition and three hours after Asian elephants were fed (3 h > fed_359.29 U/ml, Table 3, see supplement Table 6). No differences in averaged sAA activity at any time after feeding Asian elephants were observed in Kölner Zoo, Zoo Münster and Zoo Heidelberg (Fig. 1b).

**Fig. 2:**
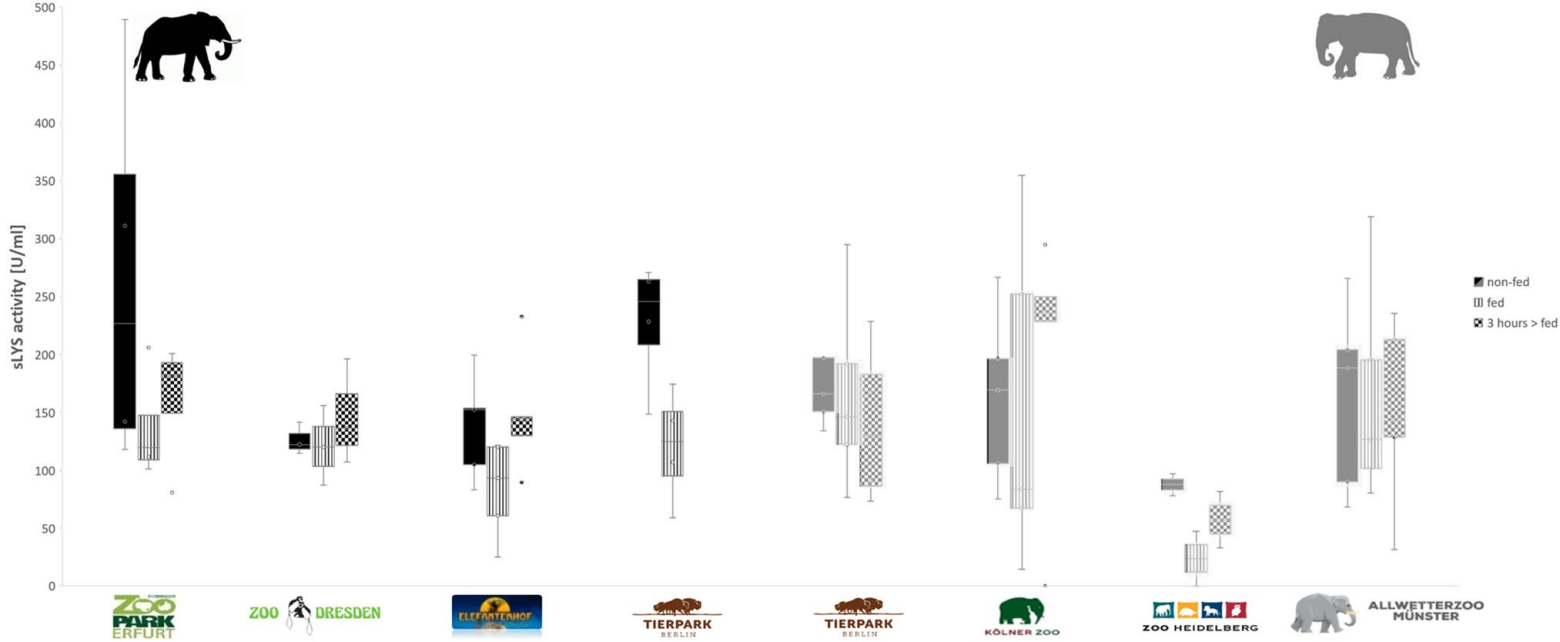
Salivary lysozyme activity of Asian and African elephants from different zoos. The boxes illustrate the 25th and 75th percentiles, bars show medians. Black filled boxes indicate saliva samples from non-fed elephants. Boxes with vertical stripes indicate saliva samples of fed elephants. Boxes with dots indicate saliva samples of elephants three hours after they were fed. The significance is displayed by *(*p* < 0.05), which illustrates a significant difference

Averaged sLYS activity of African elephants from Elefantenhof Platschow decreased significantly after African elephants were fed (non-fed: 138.64 U/ml, fed: 84.17 U/ml; Fig. 2). No changes of sLYS activity were notable three hours after feeding (Table 3, see supplement Table 6). After feeding African elephants from Tierpark Berlin and Zoopark Erfurt sLYS activity showed a decreasing trend but was not different three hours after elephants were fed in comparison to when they were non-fed or to immediately after feeding (Fig. 2, Table 3, see supplement Table 6). In Zoo Dresden, no changes of sLYS activity in African elephants were noticed at any time after feeding (Fig. 2, Table 3, see supplement Table 6). In Zoo Heidelberg, sLYS activity decreased after Asian elephants were fed which was not significantly but in comparison to immediately after feeding, within three hours after feeding sLYS activity increased significantly (Fig. 2, supplement Table 6). No changes in sLYS activity were notable in Asian and African elephants from Tierpark Berlin, and Asian elephants from Kölner Zoo and Zoo Münster at any time after they were fed (Fig. 2, supplement Table 6).

Intra-specific differences of sLYS activity within African elephants were highest in African elephants from Zoopark Erfurt when they were non-fed and in Asian elephants in Kölner Zoo after they were fed in comparison to other feeding conditions (Fig. 2, see supplement Table 4).

All African (except from Tierpark Berlin) and Asian elephants kept in Zoo Heidelberg showed no or very low sPOD activity at any sampling time, which did not change after food intake (supplement Table 6, Table 7). Feeding only yielded a decrease of sPOD activity in Asian elephants kept in Zoo Münster which increased significantly within three hours after Asian elephants were fed at the same level compared with non-fed condition of Asian elephants (Table 3, supplement Table 6). sPOD activities of Asian elephants from Tierpark Berlin and Zoo Münster were higher in comparison to other tested Asian elephants.

### (II) Influence housing condition on salivary enzyme activity

No mean differences were found in sAA activity in non-fed African elephants from different zoos (Fig. 1a, Table 4, see supplement Table 4). Mean sAA activity in Asian elephants from Tierpark Berlin was higher than in Asian elephants from Kölner Zoo (Tierpark Berlin: mean = 413.18 U/ml <> Kölner Zoo: mean = 105.54 U/ml, mean difference: 307.64 U/ml, *p* = 0.031, Fig. 1b, Table 3). Averaged sLYS and sPOD activity showed no differences within non-fed both elephant species from different zoos (Fig. 2, Table 3, see supplement Table 5). Intra-specific differences in sPOD and sAA activity were slightly lower and sLYS activity higher within African than in Asian elephants from different zoos (see supplement Table 4).

### (III) Seasonal influence on salivary enzyme activity

**Fig. 3:**
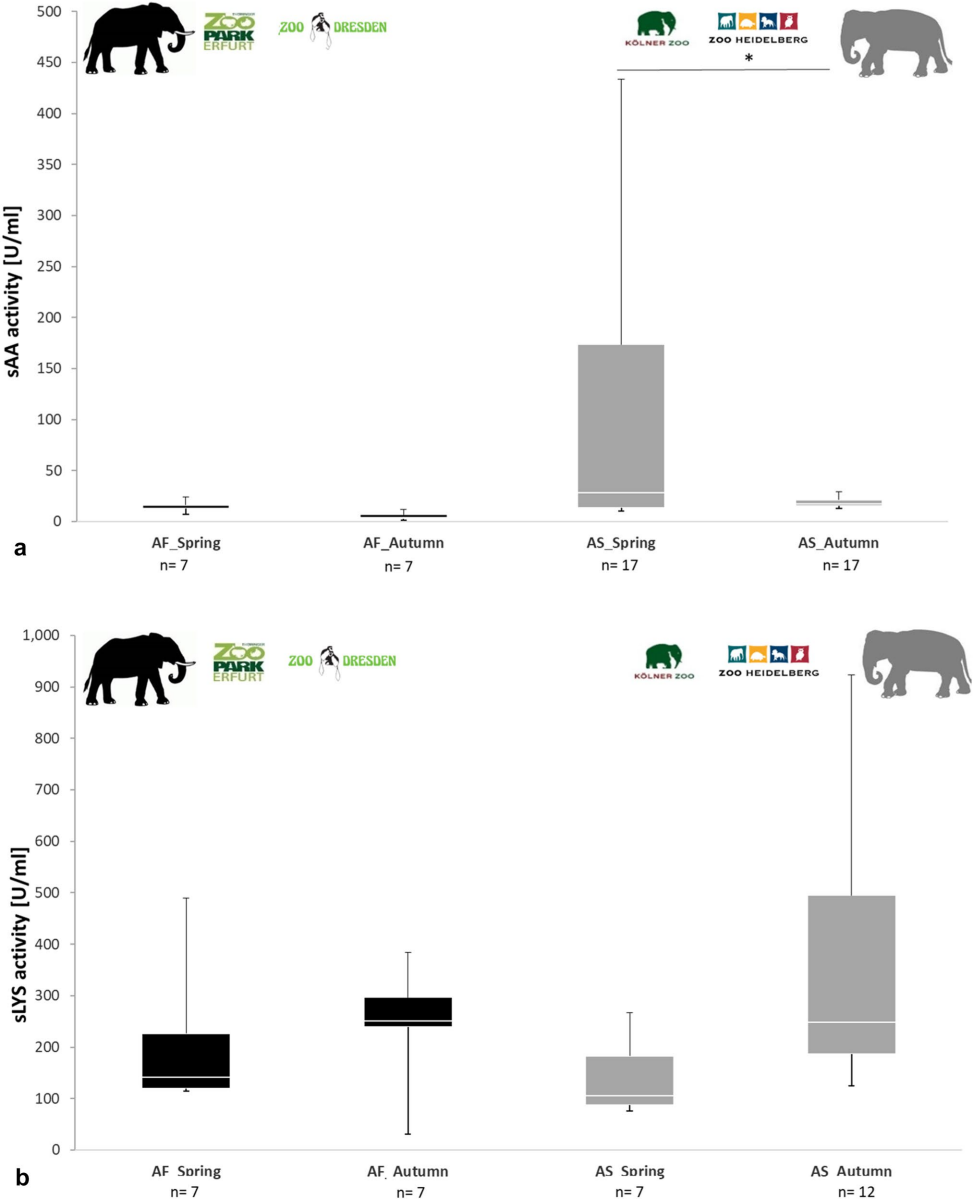
Salivary alpha amylase (sAA) and salivary lysozyme (sLYS) activity of two non-fed elephant species from four different zoos. **a** α-sAA activity (U/ml) of African elephants (AF, *Loxodonta africana*) and Asian elephants (AS, *Elephas maximus*) are shown by black and grey colored boxes, respectively. The boxes illustrate the 25th and 75th percentiles, bars show medians. The significance is displayed by *(*p* < 0.05), which illustrates a significant difference. Total sample sizes (*n* = 24): *n*_African elephants_ = 7 (Zoo Park Erfurt: *n* = 4; Zoo Dresden: *n* = 3;), n_Asian elephants_ = 17 (Kölner Zoo: *n* = 13; Zoo Heidelberg *n* = 4). **b **LYS activity (U/ml) of African elephants (AF,*Loxodonta africana*) and Asian elephants (AS,*Elephas maximus*) are shown by black and grey colored boxes, respectively. The boxes illustrate the 25th and 75th percentiles, bars show medians, dots show outlier. Total sample sizes (*n* = 24): *n*_African elephants_ = 7 (Zoo Park Erfurt: *n* = 4; Zoo Dresden: *n* = 3), n_Asian elephants-Spring_ = 7 (Kölner Zoo: *n* = 5; Zoo Heidelberg *n* = 2) *n*_Asian elephants-Autumn_ = 12 (Kölner Zoo: *n* = 8; Zoo Heidelberg: *n* = 4)

Averaged amylase activity in saliva from Asian elephants, which was collected in Spring, was higher than in saliva collected in Autumn (Spring_AS = 218.44 U/ml <> Autumn_AS = 19.45 U/ml; *p* = 0.016; mean difference = 82.69 U/ml, Fig. 3a, Table 3, see supplement Table 9). No differences were found in averaged sAA activity in African elephants collected in Spring and Autumn from Zoo Dresden and Zoopark Erfurt (Spring_AF = 14.50 U/ml <  > Autumn_AF = 5.063 U/ml; *p* = 1; mean difference = 9.438 U/ml; Fig. 3a, Table 3, see supplement Table 9). Intra-specific differences in averaged sAA activity were higher in Asian elephants collected in Spring than in Autumn from Kölner Zoo and Zoo Heidelberg (AS_Spring: 78.88—433.30 U/ml; AS_Autumn: 12.62–29.18 U/ml; Table 9).

No differences were found in averaged sLYS activity in African elephant species collected in Spring and Autumn from Zoo Dresden and Zoopark Erfurt (Spring_AF = 205.51 U/ml <> Autumn_AF = 248.37 U/ml; *p* = 0.997, Table 3), but there was a trend notable for Asian elephants, which revealed slightly higher averaged LYS activity in the saliva which was collected in Autumn in comparison to Spring (Spring_AS = 141.25 U/ml <> Autumn_AS = 353.31 U/ml; *p* = 0.076; Fig. 3b, Table 3, see supplement Table 8, Table 9). Equally, no differences were found in averaged sPOD activity in both elephant species collected in Spring and Autumn from four zoos (Spring_AF = 0.111 U/ml <> Autumn_AF = 0.090 U/ml; *p* = 1; Spring_AS = 6.719 U/ml <> Autumn_AS = 0.217 U/ml; *p* = 0.195, Table 3, see supplement Table 8, Table 9).

## Discussion

This study showed that amylase and lysozyme can be measured in Asian and African elephant saliva in an active conformation. Furthermore, irrespective of season most elephants of both species showed a lack or low salivary peroxidase activity, which was shown before (Boehlke et al. 2016). Variations in sPOD activity were only observed in two zoos possibly related to diet, which might contain peroxidase (Caligiore et al. 1982), increases sPOD activity by flavonoids (Gau et al. 2018) or interfere with sPOD (Hannig et al. 2008). High urea concentrations were found in African elephant saliva, which were suggested to serve as an energy source for bacteria and protozoa living in the oral cavity (Raubenheimer et al. 1988). Therefore, the antibacterial enzyme possibly is less advantageous in elephant saliva compared to humans, because of exploiting oral bacteria for predigesting of cellulose and lignin by the herbivore (Shipley 1999).

It was shown for all zoos, sAA activity of non-fed Asian elephants was higher than of non-fed African elephants, which was also true for both elephant species kept in Tierpark Berlin. As saliva samples were collected and both species were fed identically the day before, influence of diet and housing condition on sAA activity can be excluded. Results of this study reinforce previous suggestions that significantly higher sAA activity in Asian elephants compared to African elephants might be epigenetically attributed to the higher starch content in their natural diet (Boehlke et al. 2016). Salivary amylase activity in humans was found to cohere with the amount of starch in diet. A higher *AMY1* copy number was observed across different mammalian species consuming a high-starch diet in comparison to individuals consuming low starch amounts (Perry et al. 2007; Mandel et al. 2010; Pajic et al. 2019) leading to higher sAA activity. In addition, no differences in sLYS and sPOD activity were observed between Asian and African elephants concluding similar basic sLYS and sPOD activity levels in both species, respectively.

Sex of elephants of both species had no influence on any of the enzyme activities in saliva. Furthermore, older non-fed Asian elephants showed higher sAA activity independent from season in contrast to African elephants where age was not correlated with activities of any of the enzymes. Increasing sAA activity with age was already shown in human saliva, which was speculatively caused by age-related histological changes in acinar and ductal cells (Ben-Aryeh et al. 1990; Wang and Woolfolk 1990).

### Influence of feeding condition on salivary enzyme activity

During all investigations, salivary enzyme activities in African elephants were continuously less influenced than in Asian elephants. Contrary to the prediction (1), investigation of feeding impact on sAA, sLYS and sPOD activity revealed that feeding elephants of both species showed only influence on enzymes in Asian and African elephants kept in two of eight zoos. sAA, sLYS and sPOD activity decreased significantly after they were fed and increased slowly within three hours to the level when they were not fed. The same trend was observed in further two zoos for sAA and sLYS activity, respectively. Probably, diet, i. e. mineral powder and pellets, which were fed to both elephant species in these zoos, might have a decreasing effect on enzyme activities. As mentioned, polyphenols, such as tannins, might have influenced enzyme activities (Butler 1989; Kandra et al. 2004; Serrano et al. 2009). Asian and African elephants fed a mixture of pellets, mineral powder and cereal-based nutritional components before the second saliva collection. Therefore, it is still difficult to conclude which component might have influenced enzyme activities. Furthermore, it is still unknown how long effects of diet, which was given after the first saliva collection, can affect salivary enzyme activities. Dilution of saliva due to generally offered diet resulting in observed decrease of enzyme activity can rather be excluded because this effect was observed in only a quarter of the zoos where specimens were kept. The increase of sAA and sLYS activity after a certain time was probably caused due to swallowing of leftover food, which did not interfere with the enzymes anymore in addition to new secretion of saliva. Otherwise, effects may have been temporary and not measurable due to divergent time management during saliva collection. Although saliva was tried to collect five minutes after elephants of both species were fed, this varied sometimes until 10 minutes due to differences in individual handling of Asian and African elephants.

### Influence housing condition on salivary enzyme activity

Investigation of housing-specific influence on salivary enzyme activities in non-fed Asian and African elephants displayed no mean differences in sAA activity in non-fed African elephants from different zoos, possibly suggesting that the zoos, including diet, housing condition and saliva collection procedure, had low or comparable influence on sAA activity. Supporting this, offered diet of three zoos contained a comparable content of starch and plant material, except diet with a lower amount in Elefantenhof Platschow. In contrast, non-fed Asian elephants from Tierpark Berlin revealed slightly higher sAA activities than Asian elephants from Kölner Zoo as presumed (2). Saliva samples were collected when Asian and African elephants were not fed which excludes direct influence of diet on sAA activity. Nevertheless, differences might be caused due to zoo-specific dietary composition including different pellets, carrots and no mineral powder to both species of elephants in Tierpark Berlin in contrast to Asian elephants in Kölner Zoo. Otherwise, Asian elephants kept in both zoos originate from different habitats, i.e. Myanmar, Thailand, Sri Lanka and Singapore (Kölner Zoo) in contrast to elephants from Sumatra (Tierpark Berlin), which might affects the *AMY1* gene copy number, expression of the enzyme and/or enzyme activity as described above. In contrast to sAA activity, sLYS and sPOD activity seemed less influenceable by zoo-specific influencing factors. Intra-specific differences in enzyme activities among Asian or African elephants kept in one zoo showed that effects of feeding on salivary enzyme activities differed in intensity depending on individuality of Asian or African elephants although they were fed by identical diet. Furthermore, intra-specific differences in salivary enzyme activities within one elephant species kept in different zoos indicate individual differences that possibly influenced species-specific results.

### Seasonal influence on salivary enzyme activity

Investigation, how season influences salivary enzyme activities, revealed that sAA activity in Asian elephants from Zoo Heidelberg and Kölner Zoo was higher and sLYS activity was lower in Spring than in Autumn, which was assumed (3). On the one hand, enzyme activities might be influenced by composition of Asian elephant’s diet, which varied seasonally in both zoos. In Spring, alfalfa hay and hay from warm season grasses from past year was offered to Asian elephants containing high starch content. Low starch content branches, e. g. eucalyptus, and less dried wheat rolls than in Spring were fed to Asian elephants in Autumn. Furthermore, different content of tannins which depends on maturity grade of fed plants might interfere with enzymes (Wrangham and Waterman 1983). The bark of eucalyptus was shown to contain several plant secondary metabolites, e.g. approximately 11% up to 26% tannins (Varela et al. 2015), and formylated phloroglucinol compounds (Santos et al. 2019), which possibly effected salivary enzyme activities of Asian elephants, measured in Autumn in Köln Zoo. As mentioned, oak also contains high contents of tannins; especially up to 47.2% were detected in dry *Quercus robur* common oak gall-apples (Paaver et al. 2010). Due to high tannin content, salivary enzyme activities might have been effected by the consumption of oak in Autumn in Köln and Münster Zoo compared to Spring, as it was shown for Oak branches to interfere with sLYS activity (Green 1995). Furthermore, ginger was offered to Asian elephants in Spring, which was shown to increase sAA concentration (Cichoke 1999). Sample size of Asian elephants from Kölner Zoo were three fold higher than in Zoo Heidelberg. Therefore, seasonal variations of diet observed in Kölner Zoo might had an impact on enzyme activities. sAA and sLYS activity in African elephants from Zoo Dresden and Zoopark Erfurt showed no differences in Spring in comparison to Autumn although nutritional components differed between both zoos. Therefore, it suggests that enzyme activities in saliva of African elephants kept in zoos seemed less influenced by season than in Asian elephant saliva. To verify this, enzyme activities of saliva samples from wild living Asian and African elephants collected during different seasons have to be compared. On the other hand, enzyme activities might also be affected by immune system reactions. Investigated enzymes are also known as salivary defense proteins having antimicrobial defense functions (Fábián et al. 2012), which might affect measured salivary enzyme activity.

## Conclusion

Salivary amylase and sLYS are components of Asian and African elephant saliva in an active conformation in contrast to sPOD. Higher sAA activities in Asian elephants than in African elephants might be related to higher *AMY1* copy number and starch content of their natural diet. Both elephant species showed similar basic levels of the antibacterial and less influenceable sLYS and sPOD. Salivary enzyme activities in African elephants appear less influenceable compared to Asian elephants. Diet varying between season and zoos might affect sAA and sLYS activities primarily in Asian elephants but temporary low effects suggest sufficient buffer capacity of saliva of both elephant species.

## Supplementary Information

Below is the link to the electronic supplementary material.

Supplementary file1 (DOCX 40 kb)

## Data Availability

The data generated and analyzed during the current study are available in this published article and it´s supplements.

## References

[CR1] Abell AD, Ratcliffe MJ, Gerrard J (1998). Ascorbic acid-based inhibitors of α-amylases. Bioorg Med Chem Lett.

[CR2] Adhami VM, Syed DN, Khan N, Mukhtar H (2012). Dietary flavonoid fisetin: a novel dual inhibitor of PI3K/Akt and mTOR for prostate cancer management. Biochem Pharmacol.

[CR3] Battino M, Ferreiro MS, Gallardo I (2002). The antioxidant capacity of saliva. J Clin Periodontol.

[CR4] Ben-Aryeh H, Fisher M, Szargel R, Laufer D (1990). Composition of whole unstimulated saliva of healthy children: changes with age. Arch Oral Biol.

[CR5] Björck L, Rosén C, Marshall V, Reiter B (1975). Antibacterial activity of the lactoperoxidase system in milk against pseudomonads and other gram-negative bacteria. Appl Microbiol.

[CR6] Black MJ, Brandt RB (1974). Spectrofluorometric analysis of hydrogen peroxide. Anal Biochem.

[CR7] Blanc JJ, Barnes RFW, Craig CG et al (2007) African elephant status report 2007: an update from the African elephant database. In: Occasional Paper of the IUCN Species Survival Commission. IUCN/SSC African Elephant Specialist Group

[CR8] Boehlke C, Zierau O, Hannig C (2015). Salivary amylase—the enzyme of unspecialized euryphagous animals. Arch Oral Biol.

[CR9] Boehlke C, Pötschke S, Behringer V (2016). Does diet influence salivary enzyme activities in elephant species?. J Comp Physiol B.

[CR10] Bryant JP, Provenza FD, Pastor J (1991). Interactions between Woody plants and browsing mammals mediated by secondary metabolites. Annu Rev Ecol Syst.

[CR11] Butler LG, Hemingway RW, Karchesy JJ, Branham SJ (1989). Effects of condensed tannin on animal nutrition. Chemistry and significance of condensed tannins.

[CR12] Caligiore P, Macrae FA, St John DJB (1982). Peroxidase levels in food: relevance to colorectal cancer screening. Am J Clin Nutr.

[CR13] Carpenter GH (2013). The secretion, components, and properties of saliva. Annu Rev Food Sci Technol.

[CR14] Cerling TE, Harris JM, Leakey MG (1999). Browsing and grazing in elephants: the isotope record of modern and fossil proboscideans. Oecologia.

[CR15] Cichoke AJ (1999). The complete book of enzyme therapy.

[CR16] Clauss M, Steinmetz H, Eulenberger U (2007). Observations on the length of the intestinal tract of African *Loxodonta africana* (Blumenbach 1797) and Asian elephants *Elephas maximus* (Linné 1735). Eur J Wildl Res.

[CR17] Codron J, Lee-Thorp JA, Sponheimer M (2006). Elephant (*Loxodonta africana*) diets in Kruger National Park, South Africa: spatial and landscape differences. J Mammal.

[CR18] Codron J, Codron D, Sponheimer M (2012). Stable isotope series from elephant ivory reveal lifetime histories of a true dietary generalist. Proc R Soc B Biol Sci.

[CR19] Cooper SM, Owen-Smith N (1985). Condensed tannins deter feeding by browsing ruminants in a South African savanna. Oecologia.

[CR20] Dastjerdi A, Robert C, Watson M (2014). Low coverage sequencing of two Asian elephant (*Elephas maximus*) genomes. GigaScience.

[CR21] De Boer WF, Ntumi CP, Correia AU, Mafuca JM (2000). Diet and distribution of elephant in the Maputo Elephant Reserve, Mozambique. Afr J Ecol.

[CR22] de Freitas V, Mateus N (2001). Structural features of procyanidin interactions with salivary proteins. J Agric Food Chem.

[CR23] Deshpande SS, Cheryan M, Salunkhe DK, Luh BS (1986). Tannin analysis of food products. C R C Crit Rev Food Sci Nutr.

[CR24] dos Santos BM, Zibrandtsen JFS, Gunbilig D (2019). Quantification and localization of formylated phloroglucinol compounds (FPCs) in Eucalyptus species. Front Plant Sci.

[CR25] Edwards KL, Bansiddhi P, Paris S (2019). The development of an immunoassay to measure immunoglobulin A in Asian elephant feces, saliva, urine and serum as a potential biomarker of well-being. Conserv Physiol.

[CR26] English M, Gillespie G, Ancrenaz M (2014). Plant selection and avoidance by the Bornean elephant (Elephas maximus borneensis) in tropical forest: does plant recovery rate after herbivory influence food choices?. J Trop Ecol.

[CR27] Fábián TK, Hermann P, Beck A (2012). Salivary defense proteins: their network and role in innate and acquired oral immunity. Int J Mol Sci.

[CR28] Gau J, Arnhold J, Flemmig J (2018). Reactivation of peroxidase activity in human saliva samples by polyphenols. Arch Oral Biol.

[CR29] Gheerbrant E (2009). Paleocene emergence of elephant relatives and the rapid radiation of African ungulates. Proc Natl Acad Sci.

[CR30] Green JL (1995) The use of lysozyme in winemaking: the interaction of lysozyme with wine and efficacy in preventing malolactic fermentation in Oregon Pinot noir and Chardonnay. Master Thesis Oregon State University

[CR31] Greene W, Dierenfeld ES, Mikota S (2019) A review of Asian and African Elephant gastrointestinal anatomy, physiology, and pharmacology. J Zoo Aquarium Res 7(1):1–14. 10.19227/jzar.v7i1.329

[CR32] Guy PR (1976). The feeding behaviour of elephant (*Loxodonta africana*) in the Sengwa area, Rhodesia. South Afr J Wildl Res.

[CR33] Hambrecht S, Oerke A-K, Heistermann M, Dierkes PW (2020). Diurnal variation of salivary cortisol in captive African elephants (*Loxodonta africana*) under routine management conditions and in relation to a translocation event. Zoo Biol.

[CR34] Hannig C, Attin T, Hannig M (2004). Immobilisation and activity of human α-amylase in the acquired enamel pellicle. Arch Oral Biol.

[CR35] Hannig C, Spitzmüller B, Knausenberger S (2008). Detection and activity of peroxidase in the in situ formed enamel pellicle. Arch Oral Biol.

[CR36] Humphrey SP, Williamson RT (2001). A review of saliva: normal composition, flow, and function. J Prosthet Dent.

[CR37] Ihalin R, Loimaranta V, Tenovuo J (2006). Origin, structure, and biological activities of peroxidases in human saliva. Arch Biochem Biophys.

[CR38] Illera J-C, Silván G, Cáceres S (2014). Assessment of ovarian cycles in the African elephant (*loxodonta africana*) by measurement of salivary progesterone metabolites: elephant cycle salivary progestins. Zoo Biol.

[CR39] IUCN (2008) *Elephas maximus*. In: Choudhury A, Lahiri Choudhury DK, Desai A, Duckworth JW, Easa PS, Johnsingh AJT, Fernando P, Hedges S, Gunawardena M, Kurt F, Karanth U, Lister A, Menon V, Riddle H, Rübel A, Wikramanayake E (IUCN SSC Asian Elephant Specialist Group): the IUCN red list of threatened species 2008, p e.T7140A12828813

[CR40] Joshi R, Singh R (2008). Feeding behaviour of wild Asian elephants (*Elephas maximus*) in the Rajaji National Park. J Am Sci.

[CR41] Kabigumila J (1993). Feeding habits of elephants in Ngorongoro Crater, Tanzania. Afr J Ecol.

[CR42] Kandra L, Gyémánt G, Zajácz Á, Batta G (2004). Inhibitory effects of tannin on human salivary α-amylase. Biochem Biophys Res Commun.

[CR43] Kaufman E, Lamster IB (2000). Analysis of saliva for periodontal diagnosis. J Clin Periodontol.

[CR44] Kim B-S, Chang J-Y, Kim Y-Y, Kho H-S (2015). The effects of xylitol and sorbitol on lysozyme- and peroxidase-related enzymatic and candidacidal activities. Arch Oral Biol.

[CR45] Koch PL, Heisinger J, Moss C (1995). Isotopic tracking of change in diet and habitat use in african elephants. Science.

[CR46] Laible NJ, Germaine GR (1985). Bactericidal activity of human lysozyme, muramidase-inactive lysozyme, and cationic polypeptides against *Streptococcus sanguis* and *Streptococcus faecalis*: inhibition by chitin oligosaccharides. Infect Immun.

[CR47] Lopez-Lazaro M (2009) Distribution and biological activities of the flavonoid luteolin. Mini Rev Med Chem 9(1):31—59. 10.2174/138955709787001712 10.2174/138955709787001712 19149659

[CR48] Makhabu SW, Skarpe C, Hytteborn H, Mpofu ZD (2006). The plant vigour hypothesis revisited—how is browsing by ungulates and elephant related to woody species growth rate?. Plant Ecol.

[CR49] Mäkinen KK, Söderllng EVA (1980). A Quantitative study of mannitol, sorbitol, xylitol, and xylose in wild berries and commercial fruits. J Food Sci.

[CR50] Mandel AL, Peyrot des Gachons C, Plank KL (2010). Individual differences in amy1 gene copy number, salivary α-amylase levels, and the perception of oral starch. PLoS ONE.

[CR51] Marcilla AM, Urios V, Limiñana R (2012). Seasonal rhythms of salivary cortisol secretion in captive Asian elephants (*Elephas maximus*). Gen Comp Endocrinol.

[CR52] Mau M, Südekum K-H, Johann A (2009). Saliva of the graminivorous *Theropithecus gelada* lacks proline-rich proteins and tannin-binding capacity. Am J Primatol.

[CR53] Menargues A, Urios V, Limiñana R, Mauri M (2012). Circadian rhythm of salivary cortisol in Asian elephants (*Elephas maximus*): a factor to consider during welfare assessment. J Appl Anim Welf Sci.

[CR54] Mohapatra KK, Patra AK, Paramanik DS (2013). Food and feeding behaviour of Asiatic elephant (*Elephas maximus* Linn.) in Kuldiha Wild Life Sanctuary, Odisha, India. J Environ Biol.

[CR55] Morishita Y, Iinuma Y, Nakashima N (2000). Total and pancreatic amylase measured with 2-chloro-4-nitrophenyl-4-O-β-d-galactopyranosylmaltoside. Clin Chem.

[CR56] Nater UM, Rohleder N (2009). Salivary alpha-amylase as a non-invasive biomarker for the sympathetic nervous system: current state of research. Psychoneuroendocrinology.

[CR57] Noble RE (2000). Salivary alpha-amylase and lysozyme levels: a non-invasive technique for measuring parotid vs submandibular/sublingual gland activity. J Oral Sci.

[CR58] Owen-Smith N, Chafota J (2012). Selective feeding by a megaherbivore, the African elephant (*Loxodonta africana* ). J Mammal.

[CR59] Paaver U, Matto V, Raal A (2010). Total tannin content in distinct *Quercus robur* L. galls. J Med Plants Res.

[CR60] Pajic P, Pavlidis P, Dean K (2019). Independent amylase gene copy number bursts correlate with dietary preferences in mammals. Elife.

[CR61] Perry GH, Dominy NJ, Claw KG (2007). Diet and the evolution of human amylase gene copy number variation. Nat Genet.

[CR62] Plangsangmas T, Brown JL, Thitaram C (2020). Circadian rhythm of salivary immunoglobulin A and associations with cortisol as A stress biomarker in captive Asian elephants (*Elephas maximus*). Anim (basel).

[CR64] Proctor GB, Chan K-M (1994). A fluorometric assay of peroxidase activity utilizing 2′, 7′-dichlorofluorescein with thiocyanate: application to the study of salivary secretion. J Biochem Biophys Methods.

[CR65] Pruitt KM, Adamson M (1977). Enzyme activity of salivary lactoperoxidase adsorbed to human enamel. Infect Immun.

[CR66] Purr A (1934). The influence of vitamin C (ascorbic acid) on plant and animal amylases. Biochem J.

[CR67] Raubenheimer EJ, Dauth J, Dreyer MJ, V De Vos (1988) Parotid salivary gland of the african elephant (*Loxodonta africana*): structure and composition of the saliva. J S Afr Vet Assoc 59(4):184–187 3210214

[CR68] Robbins C, Hanley T, Hagerman A (1987). Role of tannins in defending plants against ruminants: reduction in protein availability. Ecology.

[CR69] Rohland N, Malaspinas A-S, Pollack JL (2007). Proboscidean mitogenomics: chronology and mode of elephant evolution using mastodon as outgroup. PLoS Biol.

[CR70] Sales PM, Souza PM, Simeoni LA, Silveira D (2012). α-Amylase inhibitors: a review of raw material and isolated compounds from plant source. J Pharm Pharm Sci.

[CR71] Schmitt MH, Ward D, Shrader AM (2016). Incorporating secondary metabolites, tannin-binding proteins, and diet breadth into carrying-capacity models for African elephants. Ecol Model.

[CR72] Schmitt MH, Shrader AM, Ward D (2020). Megaherbivore browsers vs. tannins: is being big enough?. Oecologia.

[CR73] Schmitt MH, Shuttleworth A, Shrader AM, Ward D (2020). The role of volatile plant secondary metabolites as pre-ingestive cues and potential toxins dictating diet selection by African elephants. Oikos.

[CR74] Serrano J, Puupponen-Pimiä R, Dauer A (2009). Tannins: current knowledge of food sources, intake, bioavailability and biological effects. Mol Nutr Food Res.

[CR75] Shinitzky M, Katchalski E, Grisaro V, Sharon N (1966). Inhibition of lysozyme by imidazole and indole derivatives. Arch Biochem Biophys.

[CR76] Shipley LA (1999). Grazers and browsers: how digestive morphology affects diet selection. Grazing Behav Livestock Wildl.

[CR77] Shrader AM, Bell C, Bertolli L, Ward D (2012). Forest or the trees: at what scale do elephants make foraging decisions?. Acta Oecol.

[CR78] Steele WF, Morrison M (1969). Antistreptococcal activity of lactoperoxidase. J Bacteriol.

[CR79] Sukumar R (1989). The Asian elephant: ecology and management.

[CR80] Tchamba MN, Seme PM (1993). Diet and feeding behaviour of the forest elephant in the Santchou Reserve, Cameroon. Afr J Ecol.

[CR81] Ullrey DE, Crissey SD, Hintz HF (1997) Elephants: nutrition and dietary husbandry. Nutrition Advisory Group. http://wildpro.twycrosszoo.org/000ADOBES/Elephants/D297nutrdietEle_NAG.pdf

[CR82] Varela P, Bermúdez X, Cancela M (2015). Use of Eucalyptus bark as tannin source. Eur J Sustain Dev.

[CR83] Vivås HJ, Sæther B-E, Andersen R (1991). Optimal twig-size selection of a generalist herbivore, the moose Alces alces: implications for plant-herbivore interactions. J Anim Ecol.

[CR84] Vray B, Hoebeke J, Saint-Guillain M (1980). A new quantitative fluorimetric assay for phagocytosis of bacteria. Scand J Immunol.

[CR85] Wang Y-B, Germaine GR (1993). Effects of pH, potassium, magnesium, and bacterial growth phase on lysozyme inhibition of glucose fermentation by *Streptococcus mutans*. J Dent Res.

[CR86] Wang C-H, Woolfolk CA (1990). Salivary amylase activity of the aged. Gerontology.

[CR87] Ward D, Muller K, Shrader AM (2017). Soil fertility on granite and sedimentary soils is associated with seasonal differences in foraging by elephants. Plant Soil.

[CR88] Wilson SL, Kerley GIH (2003). Bite diameter selection by thicket browsers: the effect of body size and plant morphology on forage intake and quality. For Ecol Manage.

[CR89] Wing LD, Buss IO (1970). Elephants and forests. Wildl Monogr.

[CR90] Wrangham RW, Waterman PG (1983). Condensed tannins in fruits eaten by chimpanzees. Biotropica.

