# Supplementary material for: Nutritional influences on enzyme activities in saliva of Asian and African elephants
Source: J Comp Physiol B. 2021 Jul 7;191(5):955–70. doi: 10.1007/s00360-021-01378-6 (PMC8380575; doi:10.1007/s00360-021-01378-6)
Supplement: Supplementary file 1 — Supplementary file1 (DOCX 40 kb) [file 360_2021_1378_MOESM1_ESM.docx]

SUPPLEMENTS

Table 4 - Summary of salivary amylase (sAA), lysozyme (sLYS) and peroxidase (sPOD) activities of Asian and African elephants in Spring.

|  |  |  |  |  | U/ml | | | |
| --- | --- | --- | --- | --- | --- | --- | --- | --- |
| Species | Feeding condition | Enzyme | Zoo | Animal quantity | Mean | Standard deviation | Minimum | Maximum |
| African elephant  (*Loxodonta* *africana*) | non-fed | sAA | Elefantenhof Platschow | 5 | 28.11 | 12.18 | 15.56 | 46.95 |
|  |  |  | Tierpark Berlin | 4 | 33.11 | 18.82 | 13.23 | 53.83 |
|  |  |  | Zoo Dresden | 3 | 12.68 | 0.261 | 12.39 | 12.87 |
|  |  |  | Zoo Erfurt | 4 | 15.86 | 7.161 | 6.743 | 24.23 |
|  |  | sLYS | Elefantenhof Platschow | 5 | 138.64 | 45.76 | 83.00 | 199.71 |
|  |  |  | Tierpark Berlin | 4 | 227.53 | 55.82 | 148.50 | 270.62 |
|  |  |  | Zoo Dresden | 3 | 126.16 | 13.97 | 114.53 | 141.65 |
|  |  |  | Zoo Erfurt | 4 | 265.03 | 172.39 | 117.93 | 489.19 |
|  |  | sPOD | Elefantenhof Platschow | 5 | 0.552 | 0.857 | 0.000 | 2.047 |
|  |  |  | Tierpark Berlin | 4 | 1.295 | 2.526 | 0.000 | 5.083 |
|  |  |  | Zoo Dresden | 3 | 0.000 | 0 | 0 | 0 |
|  |  |  | Zoo Erfurt | 4 | 0.193 | 0.299 | 0 | 0.631 |
|  | fed | sAA | Elefantenhof Platschow | 5 | 14.52 | 9.377 | 5.62 | 27.14 |
|  |  |  | Tierpark Berlin | 4 | 28.82 | 14.91 | 12.83 | 47.09 |
|  |  |  | Zoo Dresden | 3 | 17.09 | 11.85 | 4.726 | 28.36 |
|  |  |  | Zoo Erfurt | 4 | 20.53 | 10.13 | 10.17 | 33.01 |
|  |  | sLYS | Elefantenhof Platschow | 5 | 84.171 | 41.27 | 25.12 | 121.52 |
|  |  |  | Tierpark Berlin | 4 | 120.93 | 49.72 | 59.00 | 174.71 |
|  |  |  | Zoo Dresden | 3 | 121.05 | 34.24 | 87.28 | 155.75 |
|  |  |  | Zoo Erfurt | 4 | 136.72 | 47.63 | 101.14 | 206.25 |
|  |  | sPOD | Elefantenhof Platschow | 5 | 0.862 | 1.367 | 0 | 3.162 |
|  |  |  | Tierpark Berlin | 4 | 2.218 | 4.436 | 0 | 8.872 |
|  |  |  | Zoo Dresden | 3 | 0 | 0 | 0 | 0 |
|  |  |  | Zoo Erfurt | 4 | 0 | 0 | 0 | 0 |
|  | 3h > fed | sAA | Elefantenhof Platschow | 5 | 25.16 | 12.03 | 11.00 | 35.88 |
|  |  |  | Zoo Dresden | 3 | 9.081 | 5.646 | 5.177 | 15.56 |
|  |  |  | Zoo Erfurt | 4 | 20.86 | 7.395 | 11.44 | 26.88 |
|  |  | sLYS | Elefantenhof Platschow | 5 | 148.46 | 52.20 | 89.69 | 232.58 |
|  |  |  | Zoo Dresden | 3 | 146.55 | 45.41 | 107.01 | 196.15 |
|  |  |  | Zoo Erfurt | 4 | 161.09 | 54.81 | 80.80 | 200.92 |
|  |  | sPOD | Elefantenhof Platschow | 5 | 0.040 | 0.088 | 0.000 | 0.198 |
|  |  |  | Zoo Dresden | 3 | 0.000 | 0.000 | 0.000 | 0 |
|  |  |  | Zoo Erfurt | 4 | 0.184 | 0.367 | 0.000 | 0.735 |
| Asian elephants  (*Elephas* *maximus*) | non-fed | sAA | Kölner Zoo | 13 | 105.54 | 138.21 | 11.19 | 433.30 |
|  |  |  | Tierpark Berlin | 5 | 413.18 | 364.18 | 8.538 | 922.99 |
|  |  |  | Zoo Heidelberg | 4 | 89.33 | 89.49 | 10.47 | 205.89 |
|  |  |  | Zoo Münster | 5 | 213.91 | 113.53 | 78.35 | 375.58 |
|  |  | sLYS | Kölner Zoo | 5 | 162.70 | 75.51 | 75.34 | 266.43 |
|  |  |  | Tierpark Berlin | 5 | 169.14 | 28.10 | 134.31 | 197.64 |
|  |  |  | Zoo Heidelberg | 2 | 87.62 | 13.48 | 78.90 | 97.16 |
|  |  |  | Zoo Münster | 5 | 163.27 | 82.35 | 68.34 | 265.34 |
|  |  | sPOD | Kölner Zoo | 12 | 4.265 | 7.500 | 0.000 | 25.61 |
|  |  |  | Tierpark Berlin | 5 | 4.033 | 4.135 | 0.000 | 9.638 |
|  |  |  | Zoo Heidelberg | 4 | 0.249 | 0.499 | 0.000 | 0.997 |
|  |  |  | Zoo Münster | 5 | 7.395 | 7.233 | 0.204 | 18.48 |
|  | fed | sAA | Kölner Zoo | 12 | 139.92 | 166.61 | 9.841 | 423.59 |
|  |  |  | Tierpark Berlin | 5 | 132.39 | 100.88 | 13.995 | 241.67 |
|  |  |  | Zoo Heidelberg | 4 | 79.41 | 74.99 | 11.568 | 159.42 |
|  |  |  | Zoo Münster | 5 | 239.83 | 137.17 | 49.62 | 357.97 |
|  |  | sLYS | Kölner Zoo | 5 | 154.45 | 143.19 | 14.20 | 354.88 |
|  |  |  | Tierpark Berlin | 5 | 166.42 | 83.06 | 76.44 | 294.82 |
|  |  |  | Zoo Heidelberg | 2 | 23.76 | 33.61 | 0.000 | 47.53 |
|  |  |  | Zoo Münster | 5 | 164.56 | 96.53 | 80.21 | 318.85 |
|  |  | sPOD | Kölner Zoo | 12 | 0.845 | 1.356 | 0.000 | 3.936 |
|  |  |  | Tierpark Berlin | 5 | 3.948 | 5.848 | 0.000 | 13.06 |
|  |  |  | Zoo Heidelberg | 4 | 0.032 | 0.063 | 0.000 | 0.127 |
|  |  |  | Zoo Münster | 5 | 0.808 | 0.893 | 0.000 | 2.168 |
|  | 3h > fed | sAA | Kölner Zoo | 13 | 134.21 | 178.07 | 8.507 | 529.70 |
|  |  |  | Tierpark Berlin | 5 | 359.29 | 255.35 | 195.16 | 739.08 |
|  |  |  | Zoo Heidelberg | 4 | 78.80 | 70.77 | 10.39 | 155.57 |
|  |  |  | Zoo Münster | 5 | 279.66 | 116.21 | 160.61 | 429.68 |
|  |  | sLYS | Kölner Zoo | 5 | 201.71 | 115.68 | 0 | 294.82 |
|  |  |  | Tierpark Berlin | 4 | 139.99 | 71.76 | 73.16 | 228.22 |
|  |  |  | Zoo Heidelberg | 2 | 57.23 | 34.60 | 32.76 | 81.691 |
|  |  |  | Zoo Münster | 5 | 150.41 | 80.27 | 31.67 | 235.54 |
|  |  | sPOD | Kölner Zoo | 12 | 0.090 | 0.124 | 0 | 0.304 |
|  |  |  | Tierpark Berlin | 4 | 2.901 | 2.285 | 0 | 5.557 |
|  |  |  | Zoo Heidelberg | 3 | 0.382 | 0.662 | 0 | 1.147 |
|  |  |  | Zoo Münster | 5 | 9.012 | 4.309 | 5.648 | 15.074 |

Table 5 - Statistical parameters for salivary amylase (sAA), lysozyme (sLYS) and peroxidase (sPOD) activity of non-fed Asian and African elephants in Spring. GT2-Hochbergs test was used. Significant differences were marked bold.

|  |  |  |  |  |  | 95% Confidence Interval | |
| --- | --- | --- | --- | --- | --- | --- | --- |
|  | Enzyme | Zoo | Zoological institution | Mean difference in U/ml | Significance | Lower Bound | Upper Bound |
| Asian Elephants  (*Elephas* *maximus*) | sAA | Kölner Zoo | Tierpark Berlin | -307.64 | 0.031 | -594.53 | -20.75 |
|  |  |  | Zoo Heidelberg | 16.21 | 1 | -295.51 | 327.93 |
|  |  |  | Zoo Münster | -108.37 | 0.854 | -395.27 | 178.52 |
|  |  | Tierpark Berlin | Zoo Heidelberg | 323.85 | 0.102 | -41.87 | 689.57 |
|  |  |  | Zoo Münster | 199.27 | 0.484 | -145.54 | 544.07 |
|  |  | Zoo Heidelberg | Zoo Münster | -124.58 | 0.903 | -490.30 | 241.14 |
|  | sLYS | Kölner Zoo | Tierpark Berlin | -6.442 | 1 | -130.14 | 117.26 |
|  |  |  | Zoo Heidelberg | 75.076 | 0.660 | -88.56 | 238.71 |
|  |  |  | Zoo Münster | -0.572 | 1 | -124.27 | 123.13 |
|  |  | Tierpark Berlin | Zoo Heidelberg | 81.52 | 0.582 | -82.12 | 245.16 |
|  |  |  | Zoo Münster | 5.871 | 1 | -117.83 | 129.57 |
|  |  | Zoo Heidelberg | Zoo Münster | -75.65 | 0.653 | -239.29 | 87.99 |
|  | sPOD | Kölner Zoo | Tierpark Berlin | 0.232 | 1 | -9.529 | 9.993 |
|  |  |  | Zoo Heidelberg | 4.016 | 0.850 | -6.572 | 14.60 |
|  |  |  | Zoo Münster | -3.130 | 0.923 | -12.89 | 6.631 |
|  |  | Tierpark Berlin | Zoo Heidelberg | 3.784 | 0.936 | -8.518 | 16.09 |
|  |  |  | Zoo Münster | -3.362 | 0.951 | -14.96 | 8.236 |
|  |  | Zoo Heidelberg | Zoo Münster | -7.146 | 0.475 | -19.45 | 5.156 |
| African Elephants  (*Loxodonta* *africana*) | sAA | Elefantenhof Platschow | Tierpark Berlin | -5.005 | 0.988 | -30.49 | 20.48 |
|  |  |  | Zoo Dresden | 15.42 | 0.461 | -12.33 | 43.17 |
|  |  |  | Zoo Erfurt | 12.242 | 0.606 | -13.25 | 37.73 |
|  |  | Tierpark Berlin | Zoo Dresden | 20.43 | 0.238 | -8.593 | 49.45 |
|  |  |  | Zoo Erfurt | 17.25 | 0.319 | -9.621 | 44.12 |
|  |  | Zoo Dresden | Zoo Erfurt | -3.180 | 0.999 | -32.20 | 25.84 |
|  | sLYS | Elefantenhof Platschow | Tierpark Berlin | -88.89 | 0.660 | -285.15 | 107.36 |
|  |  |  | Zoo Dresden | 12.48 | 1 | -201.18 | 226.14 |
|  |  |  | Zoo Erfurt | -126.39 | 0.316 | -322.64 | 69.87 |
|  |  | Tierpark Berlin | Zoo Dresden | 101.37 | 0.659 | -122.08 | 324.82 |
|  |  |  | Zoo Erfurt | -37.49 | 0.992 | -244.37 | 169.38 |
|  |  | Zoo Dresden | Zoo Erfurt | -138.86 | 0.350 | -362.31 | 84.59 |
|  | sPOD | Elefantenhof Platschow | Tierpark Berlin | -0.743 | 0.952 | -3.576 | 2.090 |
|  |  |  | Zoo Dresden | 0.552 | 0.992 | -2.532 | 3.636 |
|  |  |  | Zoo Erfurt | 0.359 | 0.999 | -2.474 | 3.192 |
|  |  | Tierpark Berlin | Zoo Dresden | 1.295 | 0.760 | -1.930 | 4.521 |
|  |  |  | Zoo Erfurt | 1.102 | 0.817 | -1.884 | 4.088 |
|  |  | Zoo Dresden | Zoo Erfurt | -0.193 | 1 | -3.419 | 3.032 |

Table 6 - Statistical parameters of salivary amylase (sAA), lysozyme (sLYS) and peroxidase (sPOD) activity of Asian and African elephants from different zoos at different feeding conditions in Spring. Significant differences were marked bold. “non-fed” indicates non-fed elephants, “fed” shows that elephants were fed immediately before saliva collection. Third saliva sample was collected three hours after elephants were fed “3h>fed”. The significance level was defined as p < 0.05.

|  |  |  |  | Wilcoxon test | |  | Paired samples t-test | | | Wilcoxon test | | |
| --- | --- | --- | --- | --- | --- | --- | --- | --- | --- | --- | --- | --- |
| Species | Zoo | Condition |  | Z | p |  | T | df | p |  | Z | p |
| African elephant (*Loxodonta* *africana*) | Elefantenhof Platschow | non-fed_fed | sLYS | -2.023 | 0.043 | sAA | 3.023 | 4 | 0.039 | sPOD | -0.730 | 0.465 |
|  |  | non-fed_3h>fed |  | -0.135 | 0.893 |  | 0.464 | 4 | 0.667 |  | -1.604 | 0.109 |
|  |  | fed_3h>fed |  | -1.483 | 0.138 |  | -3.192 | 4 | 0.033 |  | -1.604 | 0.109 |
|  | Zoopark Erfurt | non-fed_fed |  | -1.826 | 0.068 |  | -0.867 | 3 | 0.450 |  | -1.342 | 0.180 |
|  |  | non-fed_3h>fed |  | -1,461 | 0.144 |  | -2.361 | 3 | 0.099 |  | -0.447 | 0.655 |
|  |  | fed_3h>fed |  | -0.730 | 0.465 |  | -0.059 | 3 | 0.956 |  | 1.000 | 0.317 |
|  | Zoo Dresden | non-fed_fed |  | 0 | 1 |  | -0.656 | 2 | 0.579 |  | 0 | 1 |
|  |  | non-fed_3h>fed |  | 0 | 1 |  | 1.132 | 2 | 0.375 |  | 0 | 1 |
|  |  | fed_3h>fed |  | -1,069 | 0.285 |  | 1.042 | 2 | 0.407 |  | 0 | 1 |
|  | Tierpark Berlin | non-fed_fed |  | -1.826 | 0.068 |  | 0.360 | 3 | 0.743 |  | 0 | 1 |
| Asian elephant (*Elephas* *maximus*) | Zoo Heidelberg | non-fed_fed | sAA | -0.365 | 0.715 | sLYS | 1.918 | 1 | 0.306 |  | -0.447 | 0.655 |
|  |  | non-fed_3h>fed |  | -0.730 | 0.465 |  | 0.894 | 1 | 0.536 |  | -1.000 | 0.317 |
|  |  | fed_3h>fed |  | -0.365 | 0.715 |  | -47.64 | 1 | 0.013 |  | -1.000 | 0.317 |
|  | Tierpark Berlin | non-fed_fed |  | -1.753 | 0.080 |  | 0.072 | 4 | 0.946 |  | -0.135 | 0.893 |
|  |  | non-fed_3h>fed |  | -1.461 | 0.144 |  | 0.614 | 3 | 0.583 |  | -1.461 | 0.144 |
|  |  | fed_3h>fed |  | -1.461 | 0.144 |  | 0.899 | 3 | 0.435 |  | 0 | 1.000 |
|  | Kölner Zoo | non-fed_fed |  | -1.255 | 0.209 |  | 0.205 | 4 | 0.848 |  | -1.342 | 0.180 |
|  |  | non-fed_3h>fed |  | -1.223 | 0.221 |  | -0.663 | 4 | 0.544 |  | -0.447 | 0.655 |
|  |  | fed_3h>fed |  | -0.235 | 0.814 |  | -0.529 | 4 | 0.625 |  | -1.000 | 0.317 |
|  | Zoo Münster | non-fed_fed |  | -0.135 | 0.893 |  | -0.021 | 4 | 0.984 |  | -1.214 | 0.225 |
|  |  | non-fed_3h>fed |  | -0.944 | 0.345 |  | 0.471 | 4 | 0.662 |  | -0.674 | 0.500 |
|  |  | fed_3h>fed |  | -0.135 | 0.893 |  | 0.225 | 4 | 0.833 |  | -2.023 | 0.043 |

Table 7 - Summary of salivary amylase (sAA), lysozyme (sLYS) and peroxidase (sPOD) activity of individual elephants from different zoos at different feeding conditions in Spring. “non-fed” indicates non-fed elephants, “fed” shows that elephants were fed immediately before saliva collection. Third saliva sample was collected three hours after elephants were fed “3h>fed”. "-" means no saliva sample has been measured.

|  |  |  | enzyme activity (U/ml) | | | | | | | | |
| --- | --- | --- | --- | --- | --- | --- | --- | --- | --- | --- | --- |
|  |  |  | sAA | | | sLYS | | | sPOD | | |
| Species | Zoo | Elephant ID | non-fed | fed | 3 hours > fed | non-fed | fed | 3 hours > fed | non-fed | fed | 3 hours > fed |
| African elephant *(Loxodonta africana)* | Zoopark Erfurt | af1E | 16.66 | 33.01 | 18.47 | 489.19 | 127.76 | 200.92 | 0.631 | 0 | 0 |
|  |  | af2E | 24.23 | 14.88 | 26.64 | 141.95 | 111.74 | 172.34 | 0 | 0 | 0 |
|  |  | af3E | 15.82 | 24.07 | 26.88 | 311.03 | 206.25 | 190.30 | 0.143 | 0 | 0.735 |
|  |  | af4E | 6.743 | 10.17 | 11.44 | 117.93 | 101.14 | 80.804 | 0 | 0 | 0 |
|  | Zoo Dresden | af1DD | 12.39 | 4.73 | 6.511 | 122.30 | 155.75 | 196.152 | 0 | 0 | 0 |
|  |  | af2DD | 12.79 | 28.36 | 5.177 | 114.53 | 120.11 | 107.01 | 0 | 0 | 0 |
|  |  | af3DD | 12.87 | 18.19 | 15.56 | 141.65 | 87.28 | 136.50 | 0 | 0 | 0 |
|  | Elefantenhof Platschow | af1EP | 46.95 | 20.78 | 33.28 | 104.83 | 60.66 | 129.94 | 0.255 | 0 | 0 |
|  |  | af2EP | 31.87 | 27.14 | 32.48 | 153.53 | 120.11 | 146.32 | 2.047 | 3.162 | 0.198 |
|  |  | af3EP | 20.24 | 6.037 | 11.00 | 199.71 | 93.45 | 143.74 | 0.000 | 1.087 | 0 |
|  |  | af4EP | 25.92 | 5.622 | 13.18 | 152.14 | 121.52 | 89.69 | 0.460 | 0.059 | 0 |
|  |  | af5EP | 15.56 | 13.02 | 35.88 | 82.99 | 25.12 | 232.58 | 0 | 0 | 0 |
|  | Tierpark Berlin | af1TB | 53.83 | 21.56 | - | 228.22 | 174.71 | - | 0.009 | 0 | - |
|  |  | af2TB | 43.60 | 33.80 | - | 148.50 | 143.04 | - | 0.089 | 0 | - |
|  |  | af3TB | 21.79 | 47.09 | - | 270.62 | 58.97 | - | 5.083 | 8.872 | - |
|  |  | af4TB | 13.23 | 12.83 | - | 262.79 | 107.01 | - | 0.000 | 0.000 | - |
| Asian elephant *(Elephas maximus)* | Tierpark Berlin | as1TB | 8.538 | 14.00 | - | 197.64 | 192.18 | - | 0.000 | 6.654 | - |
|  |  | as2TB | 184.21 | 98.02 | 195.16 | 134.31 | 76.44 | 90.78 | 9.638 | 13.056 | 3.308 |
|  |  | as3TB | 922.99 | 241.67 | 739.08 | 150.69 | 294.82 | 228.22 | 2.033 | 0.030 | 2.739 |
|  |  | as4TB | 628.02 | 233.56 | 227.14 | 197.10 | 146.32 | 167.82 | 7.129 | 0.000 | 5.557 |
|  |  | as5TB | 322.13 | 74.69 | 275.79 | 165.98 | 122.32 | 73.16 | 1.366 | 0 | 0 |
|  | Kölner Zoo | as1K | 173.57 | 306.09 | 298.69 | 169.25 | 252.24 | 0 | 25.10 | 0 | 0 |
|  |  | as2K | 433.30 | 91.98 | 434.47 | 75.34 | 14.20 | 235.86 | 1.080 | 0.179 | 0.227 |
|  |  | as3K | 198.286 | 306.84 | 131.49 | 196.55 | 83.733 | 228.22 | 10.54 | 3.936 | 0.299 |
|  |  | as4K | 78.875 | 423.59 | 186.45 | 105.92 | 67.22 | 249.67 | 1.054 | 1.802 | 0 |
|  |  | as5K | 326.884 | 400.97 | 529.70 | 266.43 | 354.88 | 294.82 | 7.748 | 3.086 | 0 |
|  |  | as6K | 26.562 | 29.62 | 20.49 | - | - | - | 0.278 | 0 | 0 |
|  |  | as7K | 31.894 | 30.06 | 37.48 | - | - | - | 2.101 | 0.079 | - |
|  |  | as8K | 26.478 | 27.45 | 27.06 | - | - | - | 1.575 | 0.071 | 0.100 |
|  |  | as9K | 26.717 | 29.11 | 29.62 | - | - | - | 0.807 | 0.427 | 0 |
|  |  | as10K | 13.614 | - | 8.507 | - | - | - | 0.200 | - | 0.304 |
|  |  | as11K | 13.288 | 11.10 | 16.28 | - | - | - | 0.186 | 0 | 0 |
|  |  | as12K | 11.329 | 9.841 | 10.60 | - | - | - | - | 0.556 | 0.150 |
|  |  | as13K | 11.191 | 12.35 | 13.89 | - | - | - | 0 | 0 | 0 |
|  | Zoo Heidelberg | as1HD | 112.368 | 159.42 | 155.57 | 97.16 | 0 | 32.76 | 1.00 | 0 | 1.147 |
|  |  | as2HD | 205.892 | 127.142 | 121.621 | 78.089 | 47.528 | 81.691 | 0.000 | 0.000 | 0.000 |
|  |  | as3HD | 10.474 | 11.568 | 10.385 | - | - | - | 0.000 | 0.127 | - |
|  |  | as4HD | 28.577 | 19.514 | 27.630 | - | - | - | 0.000 | 0.000 | 0.000 |
|  | Zoo Münster | as1M | 176.428 | 328.578 | 241.666 | 188.574 | 101.599 | 235.538 | 18.479 | 0.899 | 6.486 |
|  |  | as2M | 375.584 | 323.972 | 160.609 | 89.903 | 126.665 | 31.666 | 8.591 | 0.000 | 15.074 |
|  |  | as3M | 78.345 | 357.973 | 372.237 | 68.343 | 195.458 | 128.849 | 1.664 | 2.168 | 5.777 |
|  |  | as4M | 166.819 | 138.999 | 194.089 | 204.193 | 318.847 | 143.044 | 0.204 | 0.975 | 5.648 |
|  |  | as5M | 272.374 | 49.620 | 429.684 | 265.342 | 80.208 | 212.929 | 8.038 | 0.000 | 12.073 |

Table 8 – Age correlations and other parameters of salivary amylase (sAA), lysozyme (sLYS) and peroxidase (sPOD) activities in non-fed Asian and African elephants in Spring and Autumn. Pearson and Spearman correlations were run. Pearson correlation coefficient is displayed by “r”. Spearman's rank correlation coefficient “*ρ*” is also displayed and addition to the probability value “p”. “N” shows the number of tested animals. The significance level was defined as p < 0.05. Significant results were marked bold.

|  |  |  |  | Enzyme activity in U/ml | | |
| --- | --- | --- | --- | --- | --- | --- |
| Season | Species | Correlation | Parameters | sAA | sLYS | sPOD |
| Spring | Asian Elephant (*Elephas* *maximus*) | Spearman | *ρ* | 0.576 | 0.232 | 0.380 |
|  |  |  | p | 0.002 | 0.370 | 0.055 |
|  |  |  | N | 27 | 17 | 26 |
|  |  |  | Mean | 218.45 | 141.25 | 6.719 |
|  |  |  | Standard deviation | 123.31 | 71.92 | 9.258 |
|  |  |  | Minimum | 78.88 | 75.34 | 0 |
|  |  |  | Maximum | 433.30 | 266.43 | 25.61 |
|  | African Elephant *(Loxodonta africana)* | Pearson | *r* | 0.166 | 0.384 | 0.164 |
|  |  |  | p | 0.539 | 0.142 | 0.554 |
|  |  |  | N | 16 | 16 | 16 |
|  |  |  | Mean | 14.50 | 205.51 | 0.111 |
|  |  |  | Standard deviation | 5.34 | 142.94 | 0.236 |
|  |  |  | Minimum | 6.74 | 114.53 | 0 |
|  |  |  | Maximum | 24.23 | 489.19 | 0.631 |
| Autumn | Asian Elephant (*Elephas* *maximus*) | Spearman | *ρ* | 0.603 | -0.221 | -0.037 |
|  |  |  | p | 0.004 | 0.411 | 0.875 |
|  |  |  | N | 21 | 16 | 21 |
|  |  |  | Mean | 19.45 | 353.31 | 0.217 |
|  |  |  | Standard deviation | 5.21 | 235.28 | 0.369 |
|  |  |  | Minimum | 12.62 | 124.93 | 0 |
|  |  |  | Maximum | 29.18 | 922.82 | 1.117 |
|  | African Elephant *(Loxodonta africana)* | Spearman | *ρ* | 0.420 | 0.114 | 0.448 |
|  |  |  | p | 0.260 | 0.770 | 0.227 |
|  |  |  | N | 9 | 9 | 9 |
|  |  |  | Mean | 5.063 | 248.37 | 0.090 |
|  |  |  | Standard deviation | 4.024 | 109.78 | 0.154 |
|  |  |  | Minimum | 1.234 | 30.29 | 0 |
|  |  |  | Maximum | 11.71 | 383.62 | 0.321 |

Table 9 - Statistical parameters for analysis of salivary amylase (sAA), lysozyme (sLYS) and peroxidase (sPOD) activity of both non-fed elephant species. GT2-Hochberg test was used. “AS” indicates Asian elephants and “AF” indicates African elephants. Significant differences were marked bold. The significance (Sig.) level was defined as p < 0.05.

|  | | | Mean Difference | Std. Error | Sig. (p) | 95% Confidence Interval | |
| --- | --- | --- | --- | --- | --- | --- | --- |
|  |  |  |  |  |  | Lower Bound | Upper Bound |
| sAA | Spring_AS | Spring_AF | 87.22 | 34.17 | 0.081 | -6.677 | 181.12 |
|  |  | Autumn_AS | 82.69 | 26.10 | 0.016 | 10.97 | 154.40 |
|  | Spring_AF | Autumn_AF | 9.438 | 40.67 | 1.000 | -102.33 | 121.20 |
|  | Autumn_AS | Autumn_AF | 13.97 | 34.17 | 0.999 | -79.93 | 107.87 |
| sLYS | Spring_AS | Spring_AF | 64.26 | 90.70 | 0.978 | -319.22 | 190.69 |
|  |  | Autumn_AS | 212.06 | 80.70 | 0.076 | -438.91 | 14.78 |
|  | Spring_AF | Autumn_AF | 42.86 | 90.70 | 0.997 | -297.81 | 212.09 |
|  | Autumn_AS | Autumn_AF | 104.94 | 80.70 | 0.724 | -121.91 | 331.78 |
| sPOD | Spring_AS | Spring_AF | 3.150 | 1.789 | 0.403 | -1.772 | 8.073 |
|  |  | Autumn_AS | 2.968 | 1.375 | 0.195 | -0.815 | 6.752 |
|  | Spring_AF | Autumn_AF | 0.020 | 2.110 | 1 | -5.786 | 5.827 |
|  | Autumn_AS | Autumn_AF | 0.202 | 1.773 | 1 | -5.080 | 4.676 |
